# Post Mortem Leukocyte Scintigraphy in Juvenile Pigs with Experimentally Induced Osteomyelitis

**DOI:** 10.1155/2017/3603929

**Published:** 2017-09-11

**Authors:** Pia Afzelius, Ole Lerberg Nielsen, Svend Borup Jensen, Aage Kristian Olsen Alstrup

**Affiliations:** ^1^Department of Diagnostic Imaging, Section of Clinical Physiology and Nuclear Medicine, Copenhagen University Hospital, North Zealand, Hillerød, Denmark; ^2^Department of Veterinary and Animal Sciences, University of Copenhagen, Copenhagen, Denmark; ^3^Department of Nuclear Medicine, Aalborg University Hospital, Aalborg, Denmark; ^4^Department of Chemistry and Biosciences, Aalborg University, Aalborg, Denmark; ^5^Department of Nuclear Medicine & PET-Centre, Aarhus University Hospital, Aarhus, Denmark

## Abstract

We have previously demonstrated that ^111^In-labeled autologous leukocyte scintigraphy is able to detect osteomyelitis in living juvenile pigs. In animal research studies, it may well be an advantage if the animals could be scanned after euthanasia. Applying traditional scanning of living animals to euthanized animals will render anaesthesia unnecessary and be ideal for obtaining good and reliable scans for the correct interpretation of imaging afterwards, since the animals do not move. The autologous leukocytes of the pigs were collected, marked with ^111^In, and reinjected into the pigs and allowed for homing to the site of infections as usual while the pigs were alive. In this study, we demonstrate that it is possible to perform SPECT/CT with ^111^In-labelled autologous leukocytes almost 24 hrs* after* euthanasia with the same detectability of osteomyelitic lesions as in living pigs (78% versus 79%). The pigs in this study had exactly the same experimental conditions as the living pigs and were examined in parallel with the living pigs except for euthanasia prior to the leukocyte scan and that no PET/CT scans were performed.

## 1. Introduction

Chronic osteomyelitis is a progressive inflammatory process caused most often by* Staphylococcus aureus (S. aureus)*, resulting in bone destruction and sequestrum formation, which may maintain the infection. The “gold standard” for the diagnosis of chronic osteomyelitis is the presence of positive bone cultures and histopathologic examination of the bone, but noninvasive diagnostic imaging is preferred in humans. We have thus previously refined a pig-model for haematogenous spread of osteomyelitis [1, 2] and used this for examining suitable tracers for better noninvasive imaging [3–5]. In the model, we injected* S. aureus* unilaterally into the femoral artery of female juvenile domestic pigs and tested various radioactive tracers to identify the most useful diagnostic imaging protocol for bone infections. This aim necessitated a rather busy scanning protocol and prolonged anaesthesia, and due to biological variance and experiment planning we had to infect more pigs that finally were anaesthetized and scanned; the surplus of pigs was euthanized prior to the scans. We, therefore, were curious to see if it was possible to do post mortem scintigraphy of the extra pigs using a tracer with a long half-life injected before euthanasia, thus leaving no pig unused and delaying the scintigraphy to a later and more convenient time.

Naturally occurring* S. aureus* infection in pigs usually manifests as sepsis, which includes development of osteomyelitis. Johansen et al. [6] demonstrated that as in children circulating microorganisms tend to start infections in the metaphyseal ends of the long bones in juvenile pigs. This is probably due to seeding of a septic embolus aided by the slow circulation in the capillary loops in the metaphyseal growth zone making juvenile pigs particularly susceptible to epiphyseal spread and arthritis of the adjacent joint.

^111^In-leukocyte scintigraphy is a diagnostic imaging tool that displays the distribution of radiolabelled autologous leukocytes in the body. Regional or whole-body planar and/or single photon emission computed tomography (SPECT) scintigraphy perhaps combined with computed tomography (CT) of specific anatomic regions can be obtained for suspected infection/inflammation. Leukocytes are separated from plasma for labelling with ^111^In. ^111^In decays by electron capture emitting two gamma photons of 173 keV and 247 keV. The physical half-life is 67 hrs. ^111^In is bound to a lipid-soluble complex that chelates metal ions. The ^111^Indium oxine complex diffuses through the cell membrane and once intracellular, the complex dissociates, and ^111^Indium binds nuclear and cytoplasmic proteins. After labelling and reinjection radiolabelled leukocytes of humans are distributed to the blood pool, lungs, and the reticuloendothelial system of the liver, spleen, bone marrow, and major blood vessels. Imaging is performed 18–24 hrs after injection when lung, blood pool, bowel, and bladder activity are not normally seen.

We have previously demonstrated by SPECT/CT of living pigs that it is possible to mark porcine leukocytes with ^111^In, that the marked porcine leukocytes were capable of homing to sites of osteomyelitic lesions, and that the biodistribution is comparable to the human biodistribution [5]. The major difference was the accumulation of activity in the lungs of juvenile pigs, which is only a transient phenomenon in healthy human lungs. The reason for this difference is that the lungs of the pigs are part of the reticuloendothelial system.

## 2. Materials and Methods

### 2.1. Pigs and the* S. aureus* Model

Five pigs, all clinically healthy, specific pathogen-free Danish landrace-Yorkshire cross-breed female pigs (approximately 20 kg) aged 8-9 weeks, were purchased from a local commercial pig farmer. All pigs received a restricted pellet diet (DIA plus FI, DLG, Denmark). The environmental conditions were 20°C, 51% relative humidity, 12 : 12 hours light cycles, and 8 air exchanges hourly. The pigs were fasted for 16 h prior to anaesthesia but had free access to tap water. After one week of acclimatization the pigs were, under propofol anaesthesia, inoculated with a suspension of the porcine strain S54F9 [7] of* S. aureus* (10^5^ colony forming units per kg in 1.0 to 1.5 mL) into the femoral artery of the right hind limb to induce osteomyelitis, as described elsewhere [1, 2].

We have previously reported that some pigs in this osteomyelitis model will develop haematogenous dissemination of* S. aureus *leading to, for example, embolic pneumonia [3, 4]. In order to reduce the frequency of these additional lesions we used animals aged 8-9 weeks and administered procaine benzyl penicillin intramuscularly as described by Alstrup et al. [2]; after onset of clinical signs, for example, limping of the right hind limb, which occurred in all pigs, the pigs were thus once injected with a single intramuscular (IM) procaine benzyl penicillin 10,000 IE/kg (Penovet, Boehringer Ingelheim, Copenhagen, Denmark). Buprenorphine (45 *μ*g/kg Temgesic (Reckitt Benckiser, Berkshire, England)) was given three times daily (7 AM, 3 PM, and 11 PM) from time of inoculation until euthanasia. One week after inoculation, the pigs had obtained a body weight of 21-22 kg (Table 1); then anaesthesia was induced, labelling of leukocytes was performed, and then the Indium-111 labelled leukocytes were reinjected. After a various time (Table 1), the pigs were euthanized with an overdose of pentobarbital (100 mg/kg IV), and finally the pigs were scanned at convenient time points (Table 1).

The study was approved by the Danish Animal Experimentation Board (number 2012-15-2934-000123). All facilities were approved by the Danish Occupational Health Surveillance.

### 2.2. Imaging

#### 2.2.1. Computed Tomography

All examinations at the Positron Emission Centre of Aarhus University Hospital were performed with an integrated PET/computed tomography (CT) system (Siemens Biograph True point 64 PET/CT, Siemens, Erlangen, Germany), one bed position spanning 21 cm. The pigs were anaesthetized with propofol, intubated (for mechanical ventilation), and placed in dorsal recumbence as described by Alstrup and Winterdahl [8]. Initially, a scout view was obtained to secure body coverage from snout to tail.

#### 2.2.2. ^111^In-Labeled Autologous Leukocytes

After the CT-scan, ^111^In oxine labelled leukocytes were prepared according to the instructions given by the manufacturer, Mallinckrodt, Pharmaceutical, Copenhagen, Denmark. The ^111^In-labelling of leukocytes included isolation of the leukocyte fraction from autologous full blood using sedimentation and centrifugation [9]. Visual inspection of the preparation searching for clumps, clots, fibrin, and platelet aggregates was performed throughout the procedure and in particular after resuspension of the pellet of cells after centrifugation. A labelling efficiency between 50% and 80% is expected in human cells in accordance with the European guidelines [9]. We have previously demonstrated that the labelling efficacy of pig leukocytes is similar [5]. Microscopic inspection and trypan blue exclusion test for cell viability were planned if the labelling efficiency was below 50%. The labelling of the leukocyte preparations and the reinjection were performed on day 6* after* inoculation (PI), that is, approximately one day before the SPECT and PET scans (day 7 PI). The injected activity of ^111^In-labeled leukocytes was 18.1–24.7 MBq.

#### 2.2.3. Scintigraphy

In almost all cases the pigs were euthanized in the morning on the day after reinjection of labelled leukocytes and placed in cold environments waiting for the transport together with the living pigs to Aalborg University Hospital as reported previously by [5]. Whole-body planar gamma imaging was then acquired by a dual-headed gamma camera in Aalborg with a medium-energy parallel-hole collimator and using the 2 energy peaks of ^111^In: 172 and 245 keV, 15% symmetrical windows, and simultaneous two-plane anterior and posterior whole-body acquisition (500.000 counts in a 256 × 256 matrix for regional and a 256 × 1024 matrix for whole body, zoom 1.0) and supplied with single photon emission computed tomography/high dose computed tomography SPECT/CT using a Symbia T16 SPECT/CT (Siemens Medical Solutions, Hoffman Estates, Illinois, USA). The data were analysed using Philips Medical Systems DICOM Brilliance TM Workspaces, Koninklijke Philips Electronics NV 2007, DA Best, Netherlands. One day prior to the SPECT/CT high dose CT was performed in the PET-Centre of Aarhus with an integrated PET/computed tomography (CT) system (Siemens Biograph True point 64 PET/CT, Siemens, Erlangen, Germany), one bed position spanning 21 cm. The pigs were placed in dorsal recumbence. Initially, a scout view was obtained to secure body coverage from snout to tail.

#### 2.2.4. Images and Interpretation

All images were evaluated by an experienced senior specialist in nuclear medicine and computed tomography. The pigs were inoculated in the right hind limb and the left hind limb served as a healthy control. The lesions seen on CT were registered and counted (Table 2) and examined for leukocyte accumulation as summarized in Table 3. The percentage of osteomyelitic lesions accumulating ^111^In-labelled leukocytes was calculated. Focal ^111^In leukocyte accumulation that was greater than adjacent or contralateral background activity and corresponded to a bone site, or more specifically to a site of increased bone radiopharmaceutical accumulation (but did not have to be of the same intensity), was registered. Very discrete leukocyte accumulation was seen in some soft tissues and is indicated by an additional number in a parenthesis but was not accepted as an accumulation. All the OM lesions were confirmed by autopsy the following day and supplied with microbiological, immunohistochemistry, and/or microscopy later on.

## 3. Results

**Figure 1:**
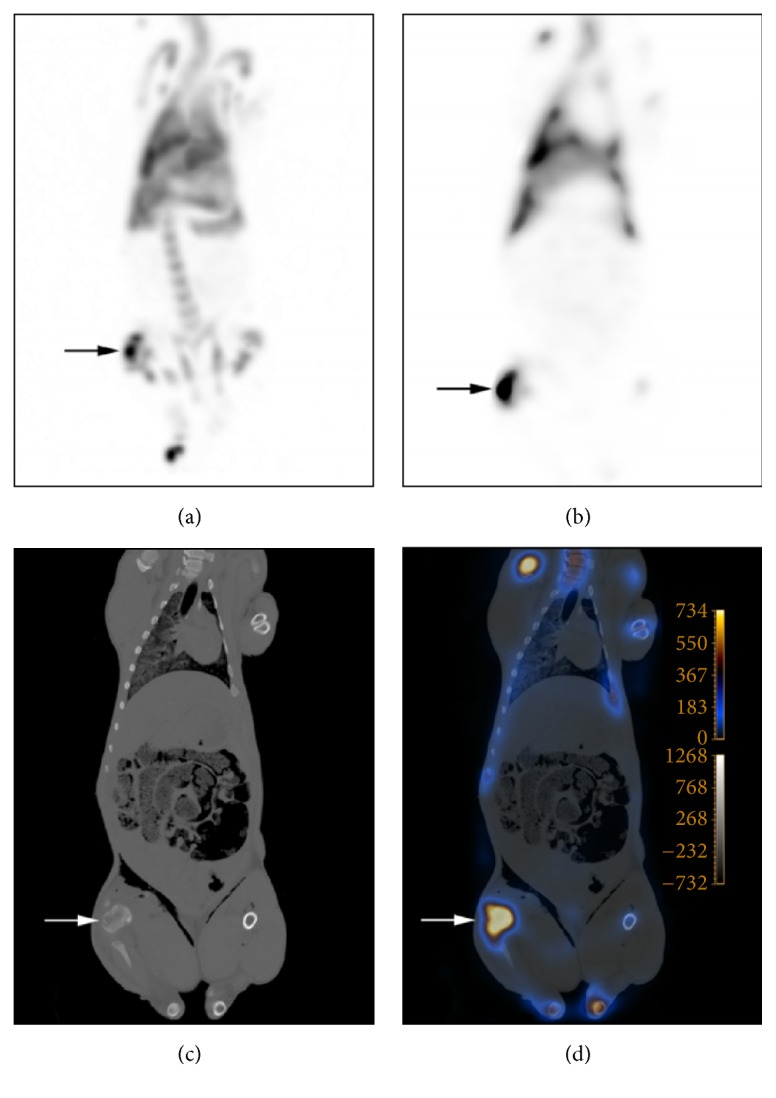
Post mortem ^111^In-leukocyte SPECT (a), ^111^In-leukocyte scintigraphy in dorsoventral projection (b), CT-scan (c) (bone window), and fused images (d) of a juvenile pig* (pig c)* demonstrating increased leukocyte accumulation in an osteomyelitic lesion in the proximal tibia, indicated by arrows, in the right hind limb. In figure (a), leukocyte accumulation is also seen in the distal femur, and the distal metatarsal bones III and IV.

The pigs were scanned 0.55–20.25 hours after euthanasia (Table 1). Diffuse leukocyte accumulation was seen in lungs, liver, and spleen. Prolonged clearance of activity from labelled leukocytes from liver and spleen was in accordance with previous results in living pigs [5]; however, the activity in the liver was lesser when scanning post mortem. As in living pigs, low excretion activity was seen in both faeces and urine. On post mortem CT the lungs were characterized by pulmonary oedema and gas effusion of variable extent was seen in parenchymal organs which are ascribed to the scintigraphy being performed post mortem. The pigs had developed foci of osteomyelitis in the inoculated limbs with insignificant signs of further spread to internal organs (Table 2). An example of ^111^In-marked autologous leukocyte accumulation in an osteomyelitic lesion visualized on post mortem scintigraphy and CT is shown in Figure 1. At autopsy, no extra osteomyelitic lesions apart from the lesions seen on CT were found.

In general, the activity of ^111^In was diffusely increased in the affected hind limb. ^111^In-labeled autologous leukocytes accumulated in 78% of the osteomyelitic lesions of the dead pigs (Table 3) which was comparable to the 79% detectability that we previously found in the living juvenile pigs [5].

## 4. Discussion

The “gold standard” for the diagnosis of chronic osteomyelitis demands invasive procedures confirming the presence of positive bone cultures and histopathologic examination of the bone, but noninvasive diagnostic imaging is preferred in humans. Animal studies can test potential treatments without confounding factors, such as prior drug use and other experiences that complicate human studies. The post mortem examination is possible and important giving a unique opportunity for confirming, for example, assumed noninvasive examination results, as imaging with potential new tracers. Our animal studies address examination of more suitable tracers for noninvasive imaging. At the end of our experiments, all animals are euthanized and the osteomyelitic foci are confirmed at autopsy, microscopically examination, and histopathological examination. We are concerned about the welfare of our research subjects. We constantly strive to minimize the risk to them; however, a surplus of animals inoculated with* S. aureus* was inevitable in our previous studies since biological models are often unpredictable and it was a necessity for us to have two animals with osteomyelitis available for the long scanning protocol. Thus, we inoculated three pigs at a time but on a few occasions experienced that none of the pigs developed osteomyelitis. In most cases, we did, however, succeed to induce two animals with osteomyelitis; sometimes even all three pigs developed lesions, and we were reluctant to “waste” one of the pigs. In this study, we demonstrated that it was possible to perform SPECT/CT with ^111^In-labelled autologous leukocytes almost 24 hrs* after* euthanasia with the same detectability of the osteomyelitic lesions as in the living pigs (78% versus 79%) by a simple count of ^111^In-labelled leukocyte accumulation in the osteomyelitic bone lesions seen on CT. Interpretation of CT, however, still requires careful scrutiny, experience, and time to detect especially small lesions.

Post mortem examination has several advantages, including that it may increase the capacity of trials with many pigs and only a single SPECT scanner is available. Also, the pigs can be euthanized at the scheduled time point and then subsequently scanned at a later time. Furthermore, it opens the possibility of comparing different scanning methods as the pigs can be euthanized immediately after the test scan without risk of continuous changes in pathology. It can also be an animal welfare advantage to scan euthanized pigs, and it does not require staff with experience in anaesthesia and monitoring of pigs. Finally, it is also an advantage that there are no motion artefacts when the pig is euthanized prior to scanning.

In short, it is possible to perform SPECT/CT with ^111^In-labelled autologous leukocytes at least up to 20 hrs after euthanasia with the same detectability of osteomyelitic lesions as in living pigs. Scanning post mortem will not hinder imaging and will result in better animal welfare since shorter anaesthesia is needed. It also makes eventual transportation easier, and more pigs can be handled at a time.

## Figures and Tables

**Table 1:** Pig characteristics and time points of scans.

Pig	Body weight (kg)	Time of CT scan the previous day Living pig	Labelling percent (%)	Injected activity (MBq)	Time points ^111^In-injection the previous day	Time points of death	Injection to death interval (h)	Time points of post mortem SPECT/CT	Death to scan interval (h)
a	21	15:26	76.8%	21.8	16:55	20:40^*∗*^	3.75	17:55	20.25
b	22	14:57	64.7%	18.1	16:20	9:00	16.67	16:49	7.82
c	22	13:14	78.8%	24.7	16:28	9:30	17.03	16:31	7.02
d	21	9:47	74.6%	24.5	16:26	16:00	23.57	16:56	0.93
e	21.5	15:05	73.8%	24.4	17:50	15:40	21.83	16:13	0.55

^*∗*^The previous day.

**Table 2:** Number of lesions defined by gross pathology, histopathology, microbiology, and/or CT in 5 juvenile euthanized pigs with haematogenous spread of *S. aureus* osteomyelitis. *S. aureus* culture or immunohistochemistry confirmed at necropsy is summarized in the lower two rows.

CT lesion	Pig				
	a	b	c^1^	d	e
Osteomyelitis	2	4	3	3	6
Sequesters	3	5	6	4	4
Osteolysis of adjacent cortical bone	2	4	6	5	4
Contiguous periosteal abscess	1	3	2	2	2
Arthritis	1^2^			0	
Hematoma/abscess at inoculation site	0	0	1	0	0
Lymph node enlargement	2	2	1	2	2

^1^Increased leukocyte accumulation in the growth zone of the left proximal calcaneus area (noninoculated limb). ^2^Acute fibrinous arthritis in the right hock joint.

**Table 3:** ^111^In-labeled leukocyte accumulation in lesions in the pelvic and right hind limb regions in 5 juvenile pigs with haematogenous *S. aureus* osteomyelitis leukocyte-scanned post mortem.

Pig	Lesion	Total number	^111^In-leukocytes
a	Osteomyelitic foci	2	1
	Contiguous periosteal abscess	1	1
	Hematoma//abscess at inoculation site	0	0
	Lymph node enlargement	2	0
	Diffusely increased accumulation in the left limb	−	+

b	Osteomyelitic foci	4	3
	Contiguous periosteal abscess	3	0
	Hematoma//abscess at inoculation site	0	−
	Lymph node enlargement	2	0

c	Osteomyelitic foci	3	3
	Contiguous periosteal abscess	2	0
	Hematoma/abscess at inoculation site	1	0
	Lymph node enlargement	1	0

d	Osteomyelitic foci	3	3
	Contiguous periosteal abscess	2	(1)
	Hematoma/abscess at inoculation site	0	−
	Lymph node enlargement	2	(1)

c	Osteomyelitic foci	6	4
	Contiguous periosteal abscess	3	3
	Hematoma/abscess at inoculation site	0	−
	Lymph node enlargement	2	0

Total	Osteomyelitic foci	18	14
	Contiguous periosteal abscess	13	4 (5)
	Hematoma/abscess at inoculation site	1	0
	Lymph node enlargement	9	(1)
